# Supplementary material for: Cloud BioLinux: pre-configured and on-demand bioinformatics computing for the genomics community
Source: BMC Bioinformatics. 2012 Mar 19;13:42. doi: 10.1186/1471-2105-13-42 (PMC3372431; doi:10.1186/1471-2105-13-42)
Supplement: Additional file 1 — Supplementary 1 Cloud BioLinux software documentation in the form of a mini, self-contained website. Users need to download and uncompress the .zip file, and open through a web browser the "index.html" file available on the main directory. (ZIP 1823 kb). [file 1471-2105-13-42-S1.ZIP › Cloud-BioLinux-Package-Documentation/Databases.html]

Bio-Linux Software Documentation Pages

Back to search form

### Databases

|  |  |
| --- | --- |
| Category | Applications |
| Databases | omssa    big-blast |
| SubCategory | Applications |
| Indexing | arb    cdbfasta    formatdb    formatrpsdb    hmmindex    makeblastdb    makembindex    sindex |
| Post\_search\_graphical | mview |
| Post\_search\_processing | mspcrunch    mview    prfx    prss |
| Retrieval | afetch    arb    blastdbcmd    cdbyank    fastacmd    hmmfetch    maxdLoad2    sfetch |
| Searching | arb    big-blast    blast+    blast2    blastall    blastcl3    blastn    blastp    blastpgp    blastx    fasta    fastf    fastm    fasts    fastx    fasty    ggsearch    glsearch    hmmpfam    hmmpfam-pvm    hmmsearch    hmmsearch-pvm    impala    maxdLoad2    megablast    netblast    omssa    pfscan    pfsearch    psiblast    rpsblast    rpsblast+    rpstblastn    seedtop    ssearch    taxblast    tblastn    tblastx    tfastf    tfastm    tfasts    tfastx    tfasty |
| Submission\_tools | sequin |
